# Bibliometric Analysis of the Top-Cited Publications and Research Trends for Stereotactic Body Radiotherapy

**DOI:** 10.3389/fonc.2021.795568

**Published:** 2021-12-03

**Authors:** Yanhao Liu, Jinying Li, Xu Cheng, Xiaotao Zhang

**Affiliations:** Department of Radiation Oncology, The Affiliated Qingdao Central Hospital of Qingdao University, Qingdao, China

**Keywords:** stereotactic body radiotherapy (SBRT), stereotactic ablative radiotherapy (SABR), bibliometric analysis, lung cancer, oligometastases, immunotherapy

## Abstract

**Objective:**

This study aims to analyze the 100 most cited papers and research trends on stereotactic body radiotherapy (SBRT).

**Methods:**

We used Web of Science to identify the 100 most frequently cited papers on SBRT on September 29, 2021 and extracted the following data: publication year, source title, country/region, organization, total citations, and average number of citations per year. The research type and research domain were classified independently by the authors. Then we carried out a bibliometric analysis to determine the trends in research on SBRT.

**Results:**

These 100 papers were cited a total of 26,540 times, and the median number of citations was 190 (range, 138-1688). “Stereotactic body radiation therapy for inoperable early stage lung cancer” by Timmerman et al. had the highest number of total citations (1688 times). International Journal of Radiation Oncology, Biology, Physics published the largest number of papers (37 papers), followed by Journal of Clinical Oncology (13 papers). The USA contributed the most papers (67 papers), followed by Canada (18 papers). Primary lung cancer (33 papers, 10,683 citations) and oligometastases (30 papers, 7,147 citations) were the most cited research areas.

**Conclusions:**

To the best of our knowledge, this is the first bibliometric analysis of the most frequently cited papers on SBRT. Our results provide insight into the historical development of SBRT and important advances in its application to cancer treatment. Early-stage non–small-cell lung cancer and oligometastases were the most cited research areas in the top 100 publications on SBRT, and SBRT combined with immunotherapy was a hot topic in the past few years. This study is helpful for researchers to identify the most influential papers and current research hotspots on SBRT.

## Introduction

Stereotactic radiotherapy (SRT) is a noninvasive tumor treatment in which potent doses of radiation are accurately delivered to target tissues in 1 to 5 fractions *via* numerous small, highly focused beams (1). In the 20th century, SRT was first used as stereotactic radiosurgery to treat brain tumors. In 2000, the utility of SRT for extracranial targets was demonstrated (2); this technique was referred to as stereotactic body radiotherapy (SBRT) or stereotactic ablative radiotherapy (SABR). In the past two decades, SBRT has developed rapidly and has become an important treatment modality for various primary or metastatic carcinomas (3).

Thousands of articles on SBRT have been published in the research areas of primary or metastatic carcinomas, radiobiology, and radiophysics, making it challenging for researchers to identify the most influential papers on SBRT or current research hotspots. Bibliometric analysis is a method of analyzing data and citation trends for a large body of literature that can help researchers to determine the state of a research area.

In the present study, we identified the 100 most frequently cited publications on SBRT and carried out a bibliometric analysis to determine the trends in research on this topic.

## Methods

We used Web of Science to identify the most heavily cited papers on SBRT. We selected the Science Citation Indexing Expanded database and conducted a literature search on September 29, 2021 without restrictions on publication time, language, or type. The search string was as follows: (Title = [stereotactic body OR stereotactic ablative] AND Title = [radiotherapy OR radiation OR radiation-therapy OR irradiation]) OR Title = (SBRT OR SABR). The search results were ranked by the number of times the papers were cited so as to identify the top 100 publications. We then used Web of Science to extract and analyze the following data: publication year, source title, country/region, organization, total citations, and average number of citations per year (12 times the number of citations per month).

Microsoft Excel software was used for descriptive statistical analysis and visual illustrations. The online platform (https://bibliometric.com) and VOSviewer 1.6.14 software were applied to construct the bibliographic coupling network based on journals, countries, co-authorship relations, and keywords so as to implement network visualization analysis.

Three authors independently classified the research type and domain of the 100 most frequently cited papers by reading the abstracts, and if needed, the articles. The research type was classified as original research, review, guideline, and meta-analysis. The research domain was classified as primary lung cancer, primary prostate carcinoma, primary liver carcinoma, primary pancreatic carcinoma, spinal metastasis, oligometastases, radiobiology, radiophysics, clinical practice of SBRT, and SABR combined with immunotherapy (I-SABR).

## Results

There were 10,727 papers identified by the search string. The 100 most heavily cited papers are listed in **Supplementary Table S1**. The total number of citations for these 100 papers was 26,540, and the median number of citations was 190 (range, 138–1688). “Stereotactic body radiation therapy for inoperable early stage lung cancer” by Timmerman et al., published in *The Journal of the American Medical Association* (*JAMA*) in 2010, had the highest number of total citations (1688 times) and second highest average number of citations per year (146.78 times) (4). “Stereotactic ablative radiotherapy versus standard of care palliative treatment in patients with oligometastatic cancers (SABR-COMET): A randomized, phase 2, open-label trial” by Palma et al., published in *The Lancet* in 2019, had the highest average number of citations per year (252 times) and the sixth highest number of total citations (588 times) (5). “Outcomes of observation vs stereotactic ablative radiation for oligometastatic prostate cancer: The ORIOLE phase 2 randomized clinical trial” by Phillips et al., published in *JAMA Oncology* in May 2020, was the most recent publication (6). Of the top 10 cited papers (**Table 1**), nine were clinical trials and one was a guideline for the SBRT technique. Six of the clinical trials examined SBRT in early-stage non–small-cell lung cancer (NSCLC), two were related to metastatic carcinoma, and one pertained to locally advanced hepatocellular carcinoma.

**Table 1:** The 10 most cited papers in SBRT until 2021.

Rank	Title	Corresponding Author	Journal	Year	Total citations	Average citations per year (rank)
1	Stereotactic Body Radiation Therapy for Inoperable Early Stage Lung Cancer	Timmerman	JAMA	2010	1688	146.78 (2)
2	Excessive toxicity when treating central tumors in a phase II study of stereotactic body radiation therapy for medically inoperable early-stage lung cancer	Timmerman	J. Clin. Oncol.	2006	1009	67.64 (7)
3	Stereotactic body radiation therapy: The report of AAPM Task Group 101	Benedict	Med. Phys.	2010	949	85.62 (6)
4	Stereotactic ablative radiotherapy versus lobectomy for operable stage I non-small-cell lung cancer: a pooled analysis of two randomised trials	Chang	Lancet Oncol.	2015	818	130.88 (4)
5	Outcome in a Prospective Phase II Trial of Medically Inoperable Stage I Non-Small-Cell Lung Cancer Patients Treated with Stereotactic Body Radiotherapy	Baumann	J. Clin. Oncol.	2009	620	50.96 (12)
6	Stereotactic ablative radiotherapy versus standard of care palliative treatment in patients with oligometastatic cancers (SABR-COMET): a randomised, phase 2, open-label trial	Palma	Lancet	2019	588	252 (1)
7	Stereotactic body radiation therapy for early-stage non-small-cell lung carcinoma: four-year results of a prospective phase II study	Fakiris	Int. J. Radiat. Oncol. Biol. Phys.	2009	577	48.76 (13)
8	Multi-Institutional Phase I/II Trial of Stereotactic Body Radiation Therapy for Liver Metastases	Schefter^a^	J. Clin. Oncol.	2009	572	46.07 (14)
9	Clinical outcomes of a phase I/II study of 48 Gy of stereotactic body radiotherapy in 4 fractions for primary lung cancer using a stereotactic body frame	Nagata	Int. J. Radiat. Oncol. Biol. Phys.	2005	481	30.54 (30)
10	Sequential Phase I and II Trials of Stereotactic Body Radiotherapy for Locally Advanced Hepatocellular Carcinoma	Bujold	J. Clin. Oncol.	2013	441	52.92 (10)

a All corresponding authors except Schefter were also first authors.

### Publication Time And Type

**Figure 1:**
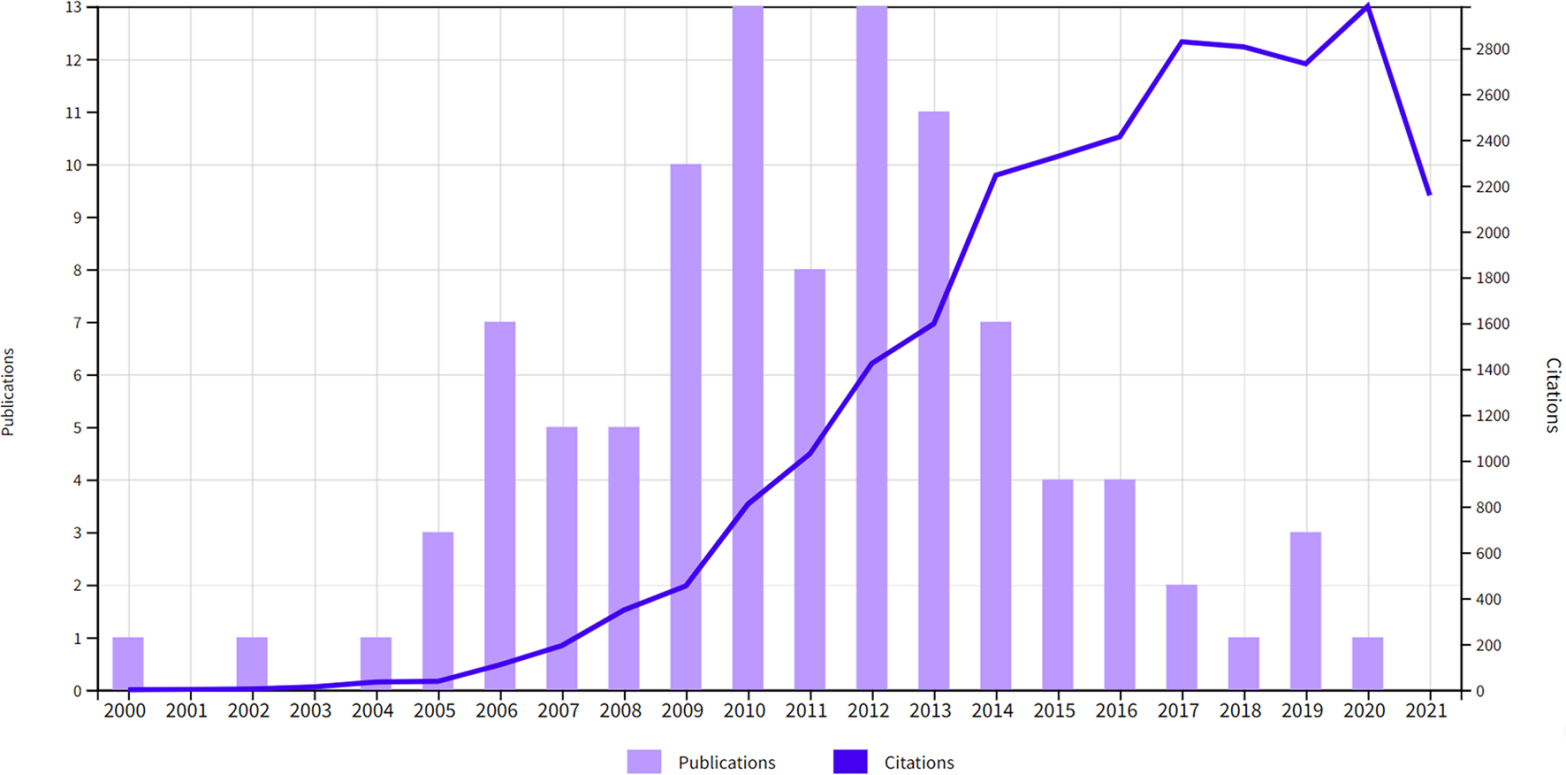
The publication time and citations distribution of the 100 most cited papers in SBRT.

These 100 most heavily cited papers were published between 2000 and 2020 (**Figure 1**). The years with the most papers in the top 100 were 2010 and 2012 (13 papers each). Among these publications, 85 were original research articles, 7 were reviews, 4 were guidelines, and 4 were meta-analyses; 45 of the 85 original research articles were clinical trials and 7 were randomized clinical trials.

### Journals

**Figure 2:**
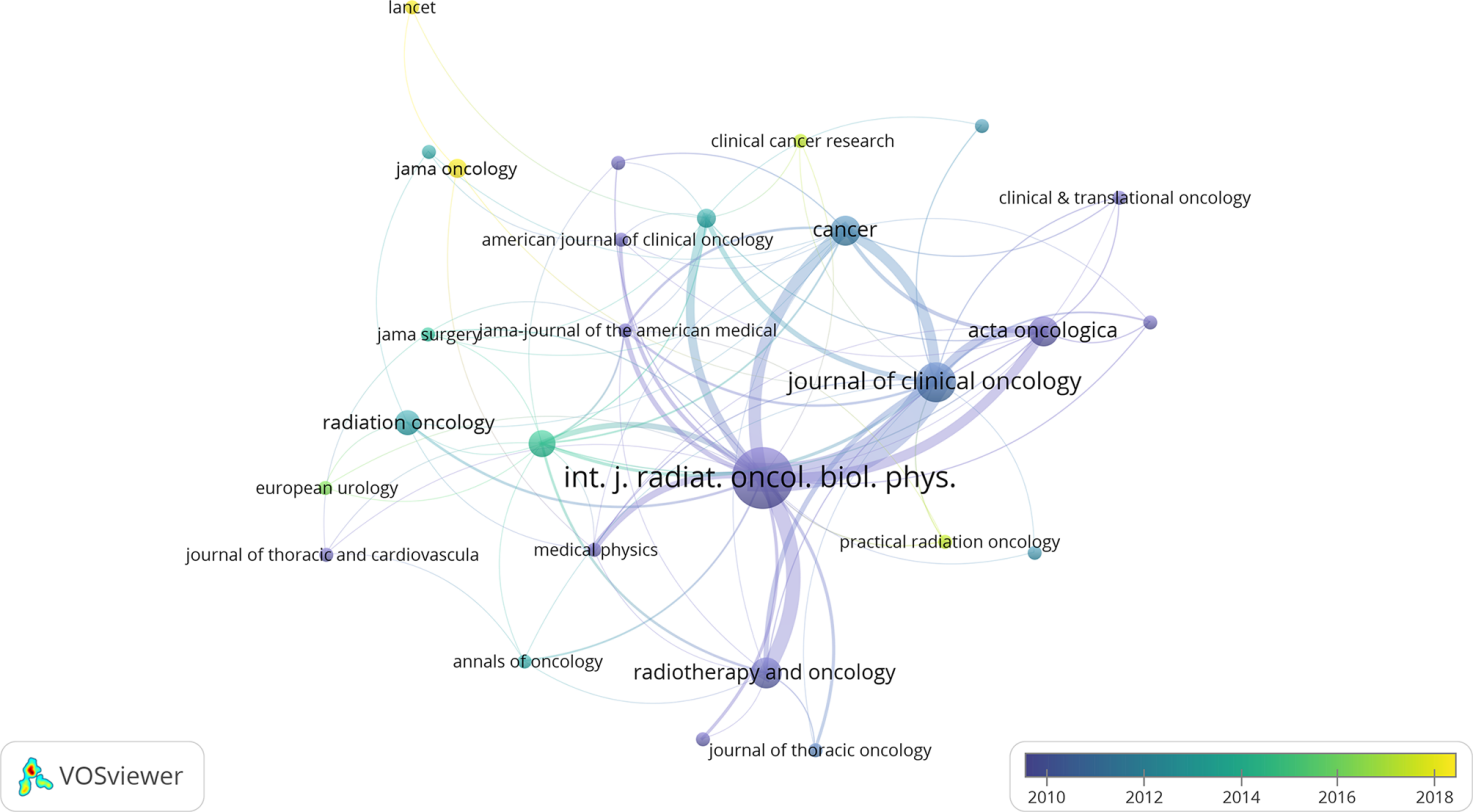
The network visualization of journals from the 100 most cited articles according to the average published year. The circle size represents the number of articles in the 100 most cited articles. The width of the curved line represents the link strength. The distance between 2 journals approximately indicates the relatedness of the nodes.

A citation network of journals was constructed based on the average published year (**Figure 2**). *International Journal of Radiation Oncology, Biology, Physics* (*IJROBP*) published the largest number of papers (37 papers), followed by *Journal of Clinical Oncology* (*JCO*; 13 papers). These two journals were at the core of the citation network. *JAMA* had the largest average number of citations per paper (one paper, 1688 citations). Among the journals that published at least two papers, *JCO* had the largest average number of citations per paper (390.54 citations).

### Countries and Institutions

**Figure 3:**
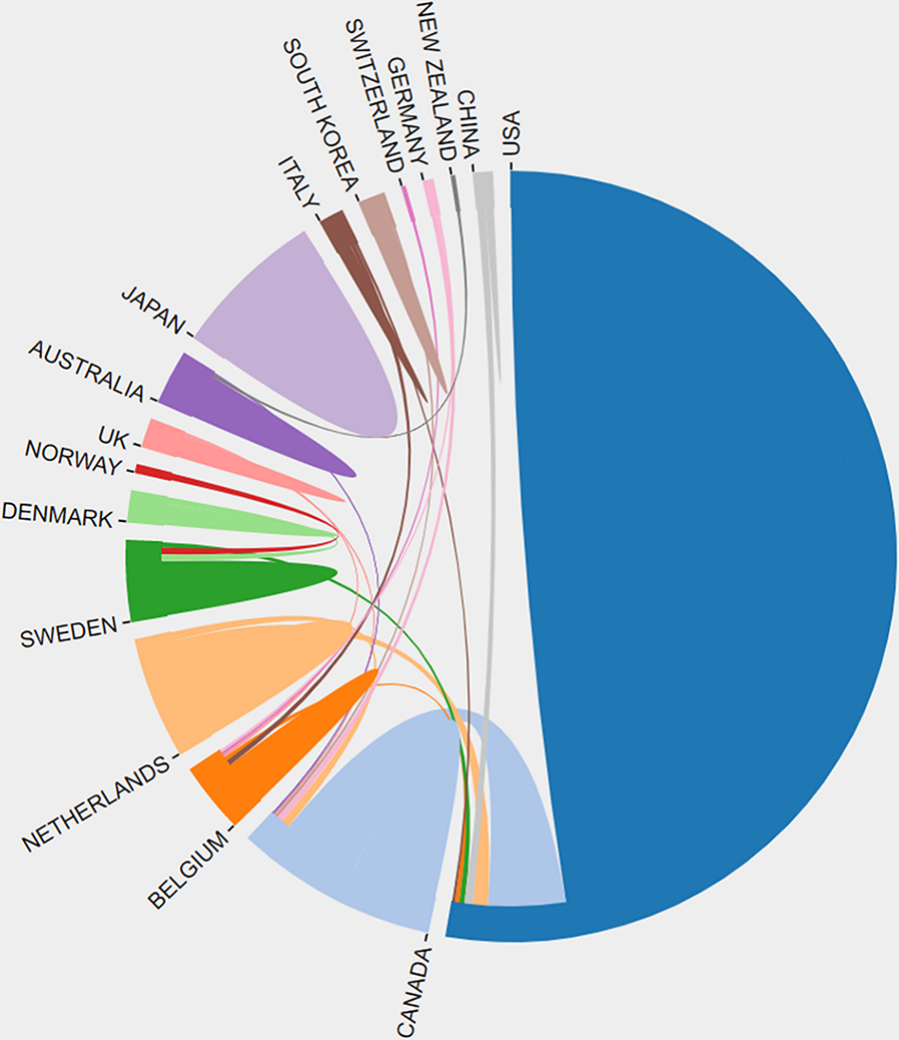
Network visualization map for countries/regions collaboration.

The authors of the 100 most cited papers were from 17 countries or regions (**Figure 3**). The United States contributed the most publications (67 papers), followed by Canada (18 papers). In terms of research institutions, University of Texas contributed the most papers (31 papers), followed by University of Texas MD Anderson Cancer Center (21 papers) (**Supplementary Figure S1**).

### Authors

**Figure 4:**
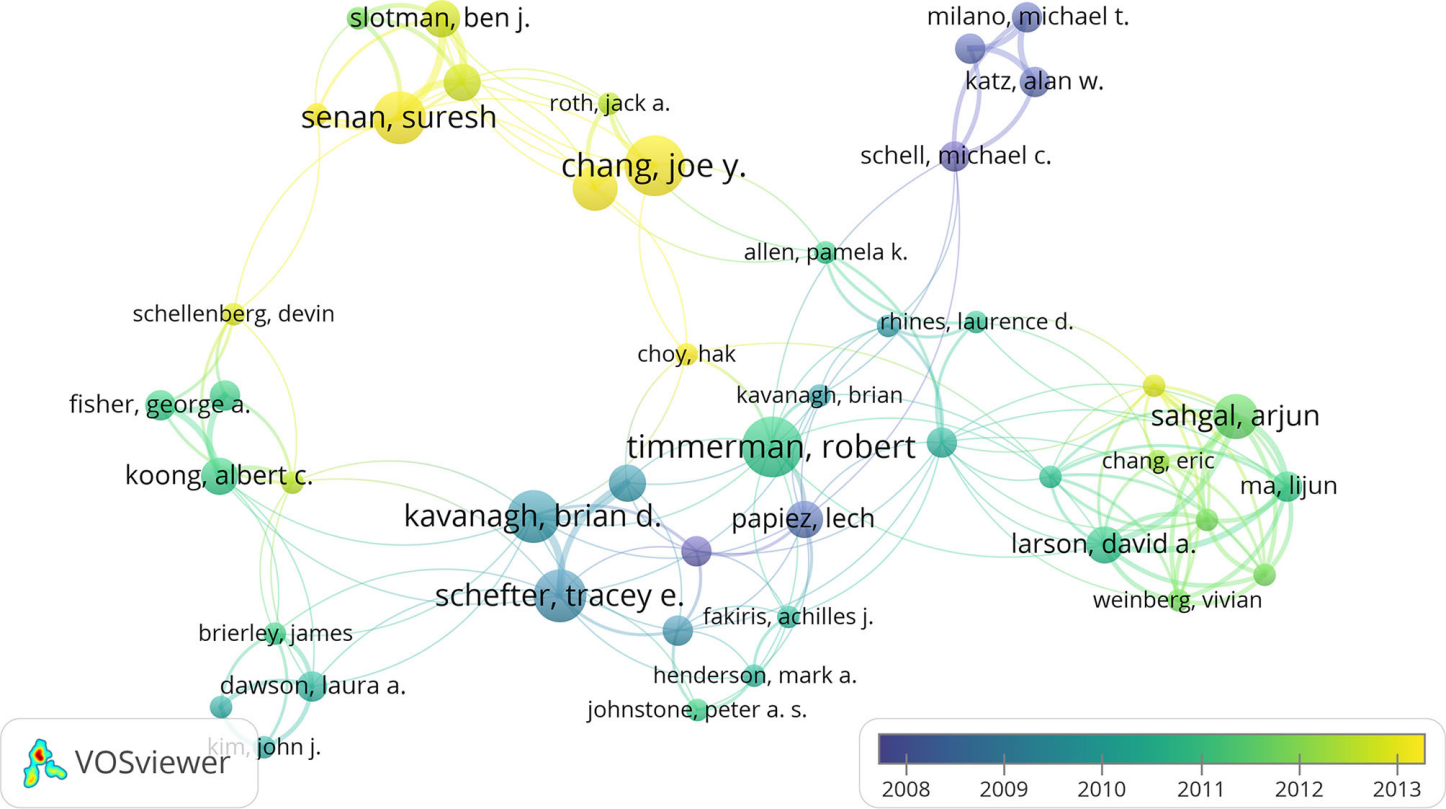
The network visualization of co-authors from the 100 most cited articles according to the average published year. The circle size represents the number of articles in the 100 most cited articles. The width of the curved line represents the link strength. The distance between 2 authors approximately indicates the relatedness of the nodes.

A network was constructed of the coauthors of the 100 most heavily cited papers (**Figure 4**). R Timmerman was at the core of this network, but new scholars that had emerged in recent years included JY Chang and S Senan. Researchers who were authors on at least five publications are shown in **Table 2**. The most prolific author was R Timmerman (15 papers), followed by JY Chang, S Senan, BD Kavanagh, and TE Schefter (eight papers each). The 15 papers by R Timmerman were cited 130 times in the top 100 most frequently cited papers.

**Table 2:** Authors of at least five of the 100 most cited papers in SBRT until 2021.

Name	Total number of most cited papers	Total citations by the most cited papers	Corresponding author frequency
Timmerman, R	15	130	5
Chang, JY	8	25	4
Senan, S	8	16	0
Kavanagh, BD	8	53	1
Schefter, TE	8	57	3
Papiez, L	6	72	0
Haasbeek, CJA	6	14	1
Komaki, R	6	16	0
Gaspar, LE	6	48	0
Larson, DA	6	24	0
Sahgal, A	6	13	6
Nagata, Y	5	41	4
Hiraoka, M	5	41	0
Slotman, BJ	5	11	0
Cardenes, HR	5	33	1
Ryu, S	5	21	0
Koong, AC	5	11	2

### Research Domains and Keywords

**Figure 5:**
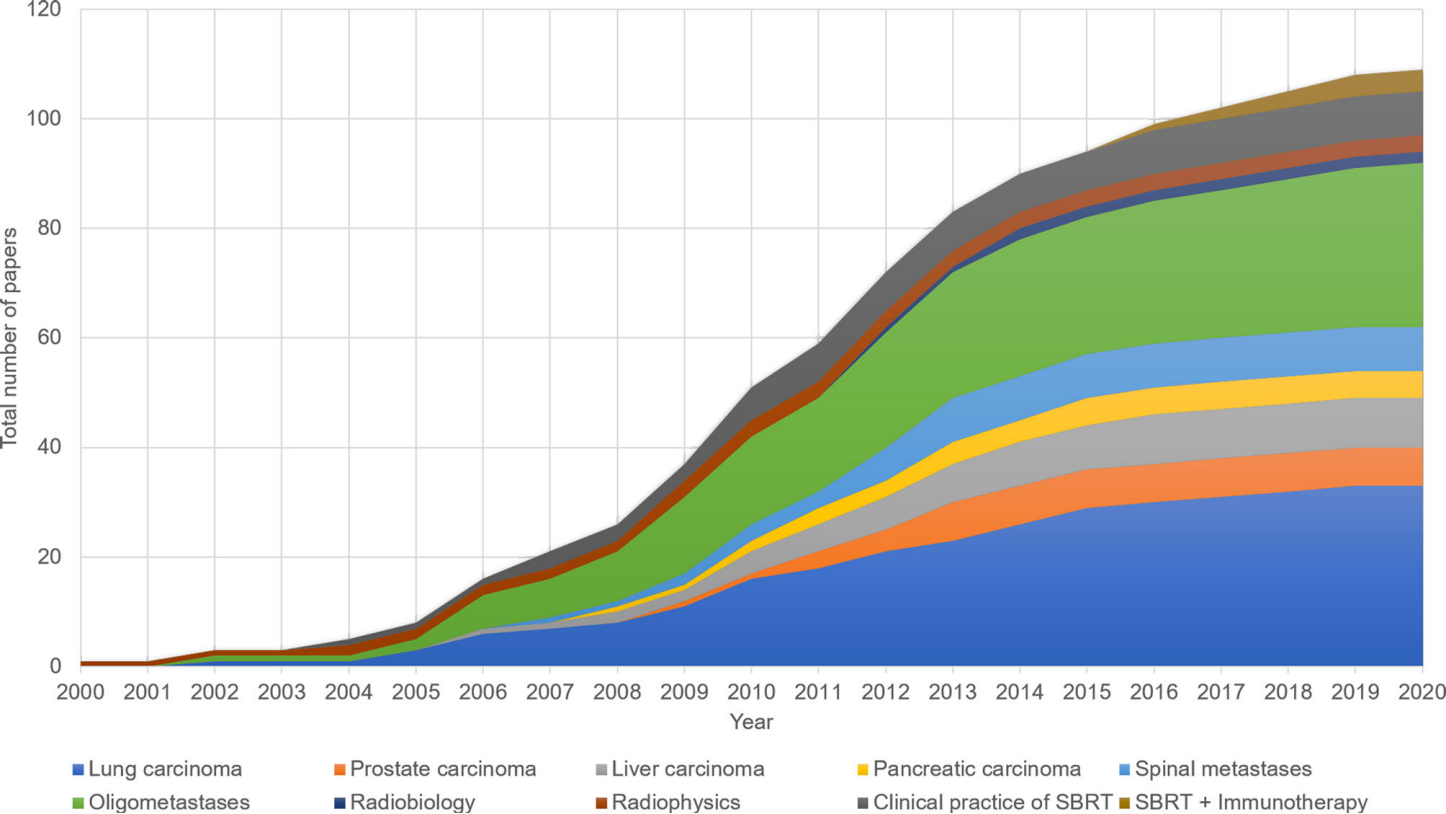
The annual publication counts of the 10 subdivided domains of SBRT.

The research domains of the 100 most cited papers are shown in **Table 3**. Most articles pertained to SBRT in the treatment of primary carcinomas (54 papers, 15,475 citations) or metastatic carcinomas (38 papers, 8562 citations). In the subdivided domain of primary NSCLC, there were 33 papers with a total of 10,683 citations (32/33 focusing on early-stage NSCLC). The trend in research domains is shown in **Figure 5**. Prior to 2005, the main research area was radiation physics, which laid the foundation for the clinical application of SBRT. From 2005 to 2010, the number of reports on the use of SBRT for the treatment of various primary or metastatic carcinomas—mainly early-stage NSCLC and oligometastases—increased. After 2010, prostate carcinoma, liver carcinoma, and spinal metastases became the research hotspots. In recent years, the most popular research areas were oligometastases and early-stage NSCLC. I-SABR is a growing research area, with four papers published on this topic between 2016 and 2019.

**Table 3:** Research domains of the 100 most cited papers in SBRT until 2021.

Research domains^a^	Number of papers	Total citations	Average citations per year (per paper)	Publication year
Primary carcinoma	54	15475	31.94	2002-2019
Lung cancer	33	10683	36.77	2002-2019
Prostate carcinoma	7	1445	21.90	2009-2013
Liver carcinoma	9	2383	27.74	2006-2016
Pancreatic carcinoma	5	964	21.71	2008-2015
Metastatic carcinoma	38	8562	30.80	2002-2020
Spinal metastasis	8	1415	17.27	2009-2013
Oligometastases^b^	30	7147	34.40	2002-2020
Radiobiology	2	600	25.66	2012-2014
Radiophysics	3	527	11.11	2000-2009
Clinical practice of SBRT	8	2395	27.43	2004-2016
SBRT + immunotherapy	4	786	63.32	2016-2019

a Some papers belonged to two domains.

b The studies about oligometastases included metastases in multiple sites.

**Figure 6:**
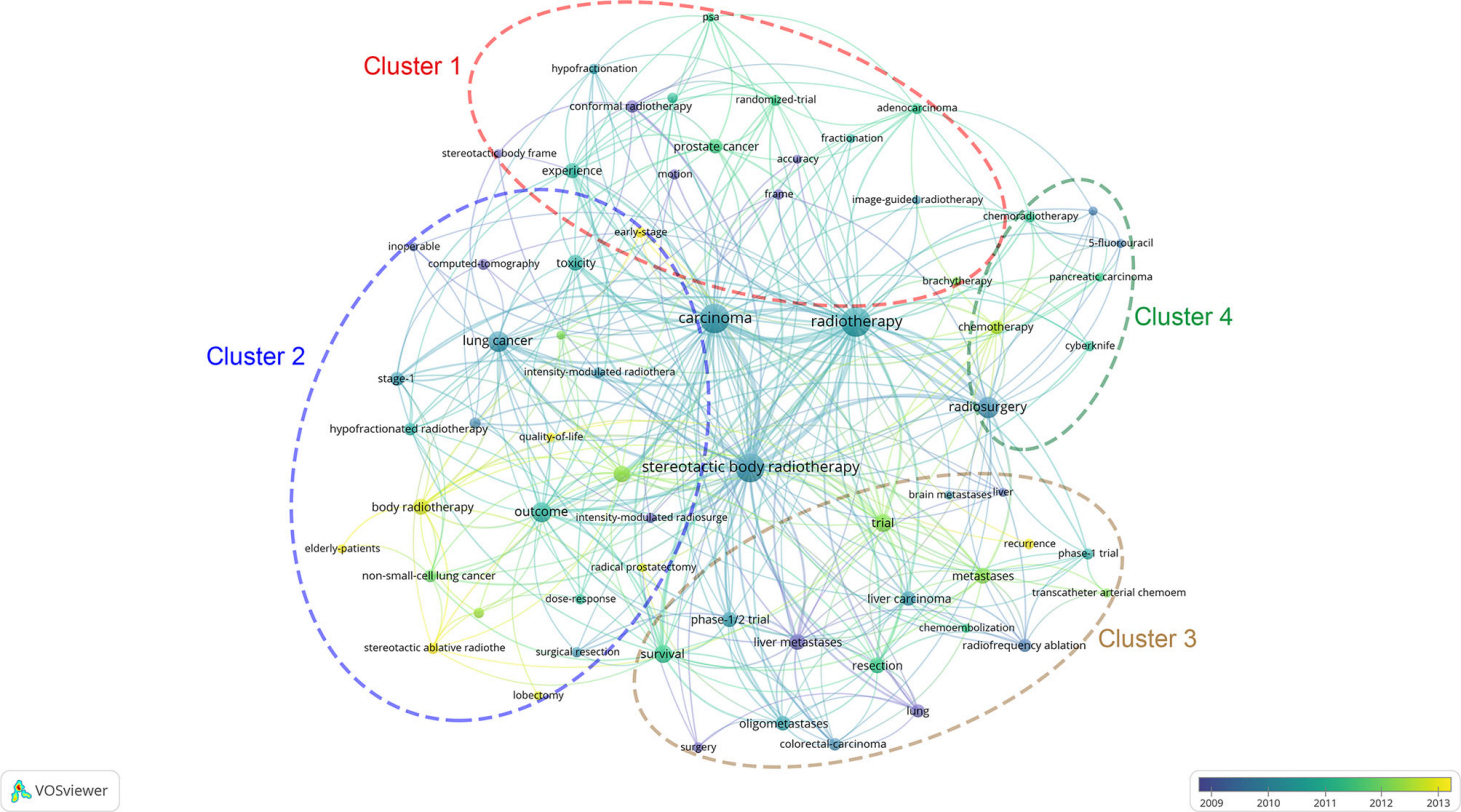
The network visualization of keywords from the 100 most cited articles according to the average published year. The circle size represents the number of articles in the 100 most cited articles. The width of the curved line represents the link strength. The distance between 2 keywords approximately indicates the relatedness of the nodes.

The keyword co-occurrence network of the 100 most frequently cited papers is shown in **Figure 6**. The top keywords in recent years were “early stage”, “quality of life”, “elderly patients”, “SABR”, “recurrence”, “metastases”, “trial”, and “chemotherapy”. Most of these keywords were related to early-stage NSCLC. The non-core keywords were classified into the following four clusters: cluster 1, prostate cancer and radiophysics (red circle in **Figure 6**); cluster 2, early-stage NSCLC (blue circle); cluster 3, metastases (brown circle); and cluster 4, chemotherapy and pancreatic carcinoma (green circle). Cluster 2 was the largest cluster.

## Discussion

Since radiation therapy has been applied to the treatment of carcinomas, radiation oncologists have attempted to deliver higher radiation doses to target tissues while minimizing toxicity. In the 1970s, after several iterations of radiotherapy technology, SRT was applied to treat brain tumors (7). After the feasibility of SBRT was verified by Wulf et al. (2), Nagata et al. reported the clinical outcomes of carcinomas treated with SBRT in 2002 (8). Since 2004, the number of studies on SBRT has grown rapidly, and has included the first guideline for the use of SBRT by the American Society for Therapeutic Radiology and Oncology (9). The use of SBRT has gradually normalized over the years, and this technology has been applied to treat early-stage NSCLC, prostate cancer, liver cancer, oligometastases, etc.

### SBRT for Early-Stage NSCLC

Of the 100 most frequently cited papers on SBRT, 32 focused on early-stage NSCLC. In 2005, Nagata et al. reported the clinical outcomes of a phase 1/2 trial of SBRT in which 45 patients with early-stage NSCLC were treated with a total dose of 48 Gy in four fractions. All tumors showed local response and no adverse events higher than grade 3 were noted (10). This was the first clinical trial of SBRT investigating the treatment of early-stage NSCLC, and had 481 citations. In 2010, Timmerman et al. reported a phase 2 clinical trial on SBRT for early-stage NSCLC; with a radiation dose of 54 Gy in three fractions, the 3-year primary tumor control rate was 97.6%, 3-year overall survival rate was 55.8%, and the grade 3/4 toxicity rate was 16.3% (4). This paper was cited 1687 times and set the standard for the treatment of inoperable NSCLC by SBRT.

In 2017, the first guidelines for SBRT in early-stage NSCLC provided recommendations on some controversial clinical issues and further standardized the clinical application of SBRT (11). The two most important questions concerning SBRT for the treatment of early-stage NSCLC were as follows: 1) the noninferiority of SBRT to surgery for operable early-stage NSCLC; and 2) patient selection criteria and dose limitation standards in the treatment of central NSCLC.

Two retrospective studies compared the clinical outcomes of SBRT and surgery for early-stage NSCLC, but obtained opposite results (12, 13). Two randomized phase 3 trials of SABR in operable stage I NSCLC (STARS and ROSEL) were terminated early on because of slow accrual, but a pooled analysis of the two trials suggested that SABR was as effective and safe as surgery for early-stage NSCLC (14). However, these studies had limitations such as small sample size and short follow-up time. In September 2021, Chang et al. reported long-term results for SABR in the treatment of operable stage I NSCLC compared to surgery (Revised STARS). The radiation dose was 54 Gy delivered in three fractions (for peripheral tumors) or 50 Gy in four fractions (for central tumors, with simultaneous integrated boost to the gross tumor totaling 60 Gy). The 3-year overall survival and severe toxicity rates of the 80 patients after SABR were 91% and 1%, respectively, which were non-inferior to the rates obtained with surgery (15).

In 2006, Timmerman et al. reported excessive toxicity when SBRT was used for early-stage NSCLC near the central airways (60–66 Gy in three fractions) (16). However, Chang et al. found that SBRT with a dose of 50 Gy in four fractions was effective and safe for centrally located early-stage NSCLC (17). Therefore, for safety reasons the doses of SBRT for central NSCLC may need to be reduced. Haasbeek et al. used SBRT with a prescription dose of 60 Gy in eight fractions to treat 63 patients with central early-stage NSCLC; four patients experienced grade 3 chest wall pain or dyspnea (18). In 2014, Chang et al. reported the use of SABR for the treatment of central NSCLC, with clinical outcomes similar to those obtained for peripheral NSCLC when normal tissue constraints were respected (19).

The abovementioned studies demonstrate that SABR is not inferior to surgery for stage I NSCLC, with comparable efficacy and safety for central and peripheral lesions when the doses delivered to normal tissue are strictly limited.

### SBRT for Oligometastases

Of the top 100 papers on SBRT, 29 focused on oligometastases, which has always been an important SBRT research area (8). Prior to 2010, most studies on SBRT for oligometastases included only patients with lung or liver metastases (20, 21). Rusthoven et al. reported that SBRT was effective and safe for liver metastases (60 Gy in three fractions) (22). In 2012, Milano et al. reported that selected patients with oligometastases treated with SBRT had good long-term survival (23). Since then, an increasing number of nonrandomized studies have confirmed the efficacy and safety of SBRT for nonspecific oligometastases (24). In 2019, the results of a randomized phase 2 study (SABR-COMET) showed that SABR improved overall survival in patients with oligometastases but 3/66 patients in the SABR group died from causes related to the treatment (5).

The optimal mode of SBRT for oligometastases has yet to be determined. A phase 3 randomized trial showed that the local recurrence rate of single-dose 24 Gy radiotherapy was lower than that 27 Gy SBRT in three fractions, with no significant difference in toxicity (25). However, the results of a phase 2 randomized trial (SAFRON II) published in August 2021 demonstrated that SABR with a single 28-Gy fraction and four fractions of 12 Gy for lung oligometastases yielded comparable outcomes (26).

The efficacy and safety of SBRT for multiple metastases is unsubstantiated. The phase 1 NRG-BR001 trial yielded preliminary evidence for the safety of SBRT for 3 to 4 metastases or 2 close metastases (27). However, it is unknown whether SBRT can provide clinical benefits in the treatment of multiple metastases.

Peter et al. reported that the most common failure after SBRT was distant metastasis (28). This not only confirmed the excellent local disease control achieved by SBRT, but also suggested that SBRT combined with systemic therapy may further improve prognosis.

To date, SBRT has been safely used for extracranial oligometastases in a variety of sites including liver, lung, and bone (29). Phase 3 trials are still needed to confirm the survival benefit and determine the optimal treatment mode and the maximum number of metastases with SABR (5).

### SBRT for Other Carcinomas

Among the 100 most frequently cited papers on SBRT, nine were focused on primary liver metastases, five on pancreatic metastases, and eight each on prostate cancer and spinal metastases. Following reports of SBRT for the treatment of liver, pancreatic, and prostate carcinoma and spinal metastases in the 2000s, clinical trials have demonstrated the efficacy and safety of SBRT in these malignancies (30–33).

Daniel et al. reported that SBRT was superior to radiofrequency ablation for hepatocellular carcinomas ≥2 cm (34), while Zhang et al. found that SBRT yielded better clinical outcomes than fractionated radiotherapy in primary liver cancer with portal vein tumor thrombus (35). A recent meta-analysis confirmed the efficacy and safety of SBRT for hepatocellular carcinoma with a regimen of 30–50 Gy in five fractions (36).

The use of SBRT in pancreatic carcinoma has not been well studied, with most trials focused on optimal dose selection (37). A recent meta-analysis indicated that SBRT did not yield better outcomes than standard therapies for locally advanced and borderline resectable pancreatic cancer (38). The efficacy and safety of SBRT combined with aggressive multiagent chemotherapy is an important research question for the future (30).

Zhao et al. confirmed the efficacy and safety of SBRT for prostate cancer based on 5-year outcomes (39). Tsang et al. compared brachytherapy in a single 19-Gy fraction, two fractions of 26 Gy, and five fractions of 36.25 Gy in prostate cancer and found that the latter two resulted in superior survival (40). Brachytherapy at a low dose rate is a standard treatment for low- and intermediate-risk prostate cancer, and a retrospective study reported that it led to comparable biochemical control and had a similar toxicity profile to SBRT at the 5-year follow-up (41).

Conventional EBRT is a standard palliative treatment for spinal metastases; however, complete response rates for pain were as low as 10%–20% (42). A phase 2/3 randomized trial recently showed that SBRT at a dose of 24 Gy in two fractions was superior to EBRT at a dose of 20 Gy in five fractions in improving the complete response rate for pain (35% vs 14%) (42).

Further studies are needed to clarify the applicability of SBRT to the treatment of liver, pancreatic, and prostate carcinoma and spinal metastases and establish the optimal regimens.

### I-SABR

Only 11 of the 100 most frequently cited papers were published after 2015, of which four were on I-SABR. In 2016, Bernstein et al. summarized the preclinical and clinical evidence for I-SABR in promoting the host antitumor immune response (43). A phase 1 trial showed that SABR combined with ipilimumab was safe and that systemic immune activation was greater after irradiation; moreover, peripheral T-cell markers could potentially predict clinical benefit (44). A phase 2 randomized trial (PEMBRO-RT) demonstrated that SABR prior to pembrolizumab treatment for locally advanced NSCLC was well tolerated, with programmed death ligand (PD-L1)–negative patients showing significantly improved prognosis (45).

Research on I-SABR has progressed rapidly. A recent phase 2 randomized trial reported that SBRT plus pembrolizumab and trametinib was effective and safe in patients with locally recurrent pancreatic cancer (46); and another phase 2 randomized trial demonstrated that neoadjuvant durvalumab combined with SBRT for early-stage NSCLC was well tolerated and associated with a high pathologic response rate (47). However, a phase 2 randomized trial of unselected patients with metastatic head and neck squamous cell carcinoma reported no improvement in response with the addition of SBRT to nivolumab (48).

I-SABR is an important subject for future research. Larger trials are necessary to investigate the molecular mechanisms underlying the effects of I-SABR, the influence of SABR on the tumor microenvironment, optimal treatment regimen, and criteria for patient selection.

### Journals, Countries, Institutions, and Authors

*IJROBP* (37 papers) and *JCO* (13 papers) published half of the 100 most frequently cited papers. Fewer articles were published in *JAMA*, *The Lancet*, and *Lancet Oncology*, but these often had a high impact. Institutions in the United States made the greatest contribution to SBRT research.

R Timmerman, who was the most influential scholar on SBRT, contributed 15 of the top 100 papers. These were published between 2004 to 2017 and were mainly focused on early-stage NSCLC and oligometastases. JY Chang has been the most prolific scholar in recent years, with most papers from his group published after 2013 and pertaining to SBRT vs. surgery for early-stage NSCLC and I-SABR. S Suresh is another new scholar who has published articles on early-stage NSCLC.

### Research Domains and Keywords

Early-stage NSCLC and oligometastases are the major focus of research on SBRT. A series of studies have established the standard of SBRT for early-stage NSCLC, and demonstrated that SBRT is not inferior to surgery. Recent studies have provided evidence for the efficacy and safety of SBRT for oligometastases, and larger clinical trials are underway. In the future, research in these two areas will be dominated by large-scale clinical trials.

I-SABR has been the most popular research domain in recent years. Immunotherapy combined with SBRT was shown to produce synergistic effects, but many unanswered questions need to be addressed in the future by basic or clinical studies.

Based on the most popular keywords, the main interests of researchers are toxicity, SBRT combined with other treatments, and evidence from clinical trials.

### Limitations

The number of citations was influenced by many factors such as publication time, research domain, and author. For example, early publications tended to have more citations. Therefore, the number of citations is not a useful metric for identifying the most influential papers. Most of the 100 papers included in our analysis were published before 2015, making it likely that some important new publications were overlooked. We used the number of citations per year to offset the impact of publication time on the most frequently cited papers, and searched important papers published in the last few years and discussed their findings in order to stay abreast of the latest advances in SBRT research.

Most of the selected papers focused on early-stage NSCLC or oligometastases, but other domains, while less popular, are also important. As we included different subdivisions in a single bibliometric analysis, it was inevitable that smaller research domains were excluded. However, we discussed the development of each domain in order to determine the research status.

We used only the Web of Science search engine to identify publications; therefore, papers in other databases or that were not in English may have been missed, which could have led to bias in citation statistics and the omission of important work.

Finally, although we tried to identify the domains and study design of each article, it was difficult to analyze these articles in more detail in a bibliometric analysis.

## Conclusion

To the best of our knowledge, this is the first bibliometric analysis of the most frequently cited papers on SBRT. Our results provide insight into the historical development of SBRT and important advances in its application to cancer treatment. Early-stage NSCLC and oligometastases were the most cited research areas in the top 100 publications on SBRT, and I-SABR was a hot topic in the past few years. This study is helpful for researchers to identify the most influential papers and current research hotspots on SBRT.

## Data Availability Statement

The raw data supporting the conclusions of this article will be made available by the authors, without undue reservation.

## Author Contributions

XZ and YL contributed to the conception of the study. YL analyzed the data. YL, JL, and XC contributed to the review of literatures. YL wrote the manuscript. All authors contributed to the article and approved the submitted version.

## Conflict of Interest

The authors declare that the research was conducted in the absence of any commercial or financial relationships that could be construed as a potential conflict of interest.

## Publisher’s Note

All claims expressed in this article are solely those of the authors and do not necessarily represent those of their affiliated organizations, or those of the publisher, the editors and the reviewers. Any product that may be evaluated in this article, or claim that may be made by its manufacturer, is not guaranteed or endorsed by the publisher.
