# Supplementary figures and images for: Bibliometric Analysis of the Top-Cited Publications and Research Trends for Stereotactic Body Radiotherapy
Source: Front Oncol. 2021 Dec 3;11:795568. doi: 10.3389/fonc.2021.795568 (PMC8677697; doi:10.3389/fonc.2021.795568)

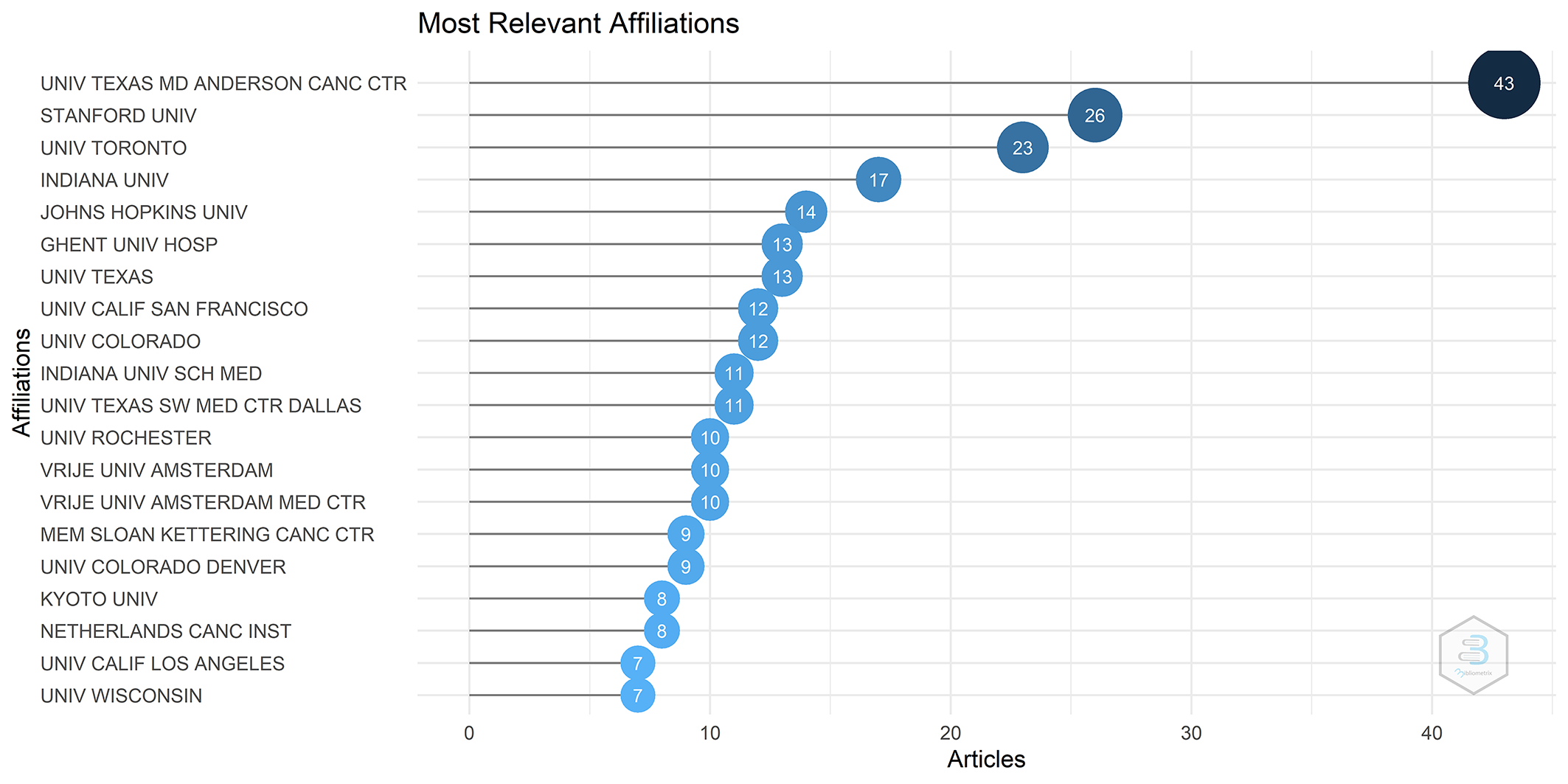

Supplement: Supplementary file 1 [file Image_1.tif]
