# Supplementary material for: Bibliometric Analysis of the Top-Cited Publications and Research Trends for Stereotactic Body Radiotherapy
Source: Front Oncol. 2021 Dec 3;11:795568. doi: 10.3389/fonc.2021.795568 (PMC8677697; doi:10.3389/fonc.2021.795568)
Supplement: Supplementary file 2 [file Table_1.docx]

|  | **TABLE S1** The 100 most cited papers in SBRT until 2021. | | | | | | | |
| --- | --- | --- | --- | --- | --- | --- | --- | --- |
| Rank | | Title | corresponding author | Journal | Year | Total citation | Average citation per year (rank) |  |
| 1 | | Stereotactic Body Radiation Therapy for Inoperable Early Stage Lung Cancer | TIMMERMAN, R | JAMA | 2010 | 1688 | 146.78 |  |
| 2 | | Excessive toxicity when treating central tumors in a phase II study of stereotactic body radiation therapy for medically inoperable early-stage lung cancer | TIMMERMAN, R | J. Clin. Oncol. | 2006 | 1009 | 67.642 |  |
| 3 | | Stereotactic body radiation therapy: The report of AAPM Task Group 101 | BENEDICT, SH | Med. Phys. | 2010 | 949 | 85.624 |  |
| 4 | | Stereotactic ablative radiotherapy versus lobectomy for operable stage I non-small-cell lung cancer: a pooled analysis of two randomised trials | CHANG, JY | Lancet Oncol. | 2015 | 818 | 130.88 |  |
| 5 | | Outcome in a Prospective Phase II Trial of Medically Inoperable Stage I Non-Small-Cell Lung Cancer Patients Treated With Stereotactic Body Radiotherapy | BAUMANN, P | J. Clin. Oncol. | 2009 | 620 | 50.959 |  |
| 6 | | Stereotactic ablative radiotherapy versus standard of care palliative treatment in patients with oligometastatic cancers (SABR-COMET): a randomised, phase 2, open-label trial | PALMA, DA | Lancet | 2019 | 588 | 252 |  |
| 7 | | STEREOTACTIC BODY RADIATION THERAPY FOR EARLY-STAGE NON-SMALL-CELL LUNG CARCINOMA: FOUR-YEAR RESULTS OF A PROSPECTIVE PHASE II STUDY | FAKIRIS, AJ | Int. J. Radiat. Oncol. Biol. Phys. | 2009 | 577 | 48.761 |  |
| 8 | | Multi-Institutional Phase I/II Trial of Stereotactic Body Radiation Therapy for Liver Metastases | SCHEFTER, TE | J. Clin. Oncol. | 2009 | 572 | 46.067 |  |
| 9 | | Clinical outcomes of a phase I/II study of 48 Gy of stereotactic body radiotherapy in 4 fractions for primary lung cancer using a stereotactic body frame | NAGATA, Y | Int. J. Radiat. Oncol. Biol. Phys. | 2005 | 481 | 30.54 |  |
| 10 | | Sequential Phase I and II Trials of Stereotactic Body Radiotherapy for Locally Advanced Hepatocellular Carcinoma | BUJOLD, A | J. Clin. Oncol. | 2013 | 441 | 52.92 |  |
| 11 | | Multi-Institutional Phase I/II Trial of Stereotactic Body Radiation Therapy for Lung Metastases | SCHEFTER, TE | J. Clin. Oncol. | 2009 | 424 | 34.148 |  |
| 12 | | STEREOTACTIC BODY RADIOTHERAPY (SBRT) FOR OPERABLE STAGE I NON SMALL-CELL LUNG CANCER: CAN SBRT BE COMPARABLE TO SURGERY? | ONISHI, H | Int. J. Radiat. Oncol. Biol. Phys. | 2011 | 399 | 40.923 |  |
| 13 | | Stereotactic body radiation therapy of early-stage non-small-cell lung carcinoma: Phase I study | MCGARRY, RC | Int. J. Radiat. Oncol. Biol. Phys. | 2005 | 375 | 23.684 |  |
| 14 | | Phase I study of individualized stereotactic body radiotherapy for hepatocellular carcinoma and intrahepatic cholangiocarcinoma | DAWSON, LA | J. Clin. Oncol. | 2008 | 374 | 27.534 |  |
| 15 | | Stereotactic body radiation therapy for primary and metastatic liver tumors: A single institution phase i-ii study | ROMERO, AM | Acta Oncol. | 2006 | 337 | 22.467 |  |
| 16 | | Phase I/II study of stereotactic body radiotherapy for spinal metastasis and its pattern of failure | CHANG, EL | J. Neurosurg.-Spine | 2007 | 327 | 23.219 |  |
| 17 | | Stereotactic body radiotherapy for oligometastases | TREE, AC | Lancet Oncol. | 2013 | 322 | 37.154 |  |
| 18 | | Phase I Study of Individualized Stereotactic Body Radiotherapy of Liver Metastases | DAWSON, LA | J. Clin. Oncol. | 2009 | 317 | 25.53 |  |
| 19 | | Oligometastases Treated With Stereotactic Body Radiotherapy: Long-Term Follow-Up of Prospective Study | MILANO, MT | Int. J. Radiat. Oncol. Biol. Phys. | 2012 | 308 | 33.6 |  |
| 20 | | The Tumor Radiobiology of SRS and SBRT: Are More Than the 5 Rs Involved? | BROWN, JM | Int. J. Radiat. Oncol. Biol. Phys. | 2014 | 307 | 40.484 |  |
| 21 | | Patterns of disease recurrence after stereotactic ablative radiotherapy for early stage non-small-cell lung cancer: a retrospective analysis | LAGERWAARD, FJ | Lancet Oncol. | 2012 | 307 | 33.798 |  |
| 22 | | Stereotactic body radiation therapy in multiple organ sites | TIMMERMAN, RD | J. Clin. Oncol. | 2007 | 306 | 21.103 |  |
| 23 | | Stereotactic body radiotherapy for localized prostate cancer: Pooled analysis from a multi-institutional consortium of prospective phase II trials | KING, CR | Radiother. Oncol. | 2013 | 297 | 37.915 |  |
| 24 | | Phase II study on stereotactic body radiotherapy of colorectal metastases | HOYER, M | Acta Oncol. | 2006 | 297 | 19.8 |  |
| 25 | | Radiation-Induced Vascular Damage in Tumors: Implications of Vascular Damage in Ablative Hypofractionated Radiotherapy (SBRT and SRS) | SONG, CW | Radiat. Res. | 2012 | 293 | 30.842 |  |
| 26 | | A Phase I trial of stereotactic body radiation therapy (SBRT) for liver metastases | SCHEFTER, TE | Int. J. Radiat. Oncol. Biol. Phys. | 2005 | 288 | 17.907 |  |
| 27 | | LONG-TERM OUTCOMES FROM A PROSPECTIVE TRIAL OF STEREOTACTIC BODY RADIOTHERAPY FOR LOW-RISK PROSTATE CANCER | KING, CR | Int. J. Radiat. Oncol. Biol. Phys. | 2012 | 275 | 28.696 |  |
| 28 | | STEREOTACTIC BODY RADIOTHERAPY FOR PRIMARY HEPATOCELLULAR CARCINOMA | ANDOLINO, DL | Int. J. Radiat. Oncol. Biol. Phys. | 2011 | 267 | 27.153 |  |
| 29 | | AMERICAN SOCIETY FOR THERAPEUTIC RADIOLOGY AND ONCOLOGY (ASTRO) AND AMERICAN COLLEGE OF RADIOLOGY (ACR) PRACTICE GUIDELINE FOR THE PERFORMANCE OF STEREOTACTIC BODY RADIATION THERAPY | POTTERS, L | Int. J. Radiat. Oncol. Biol. Phys. | 2010 | 266 | 22.964 |  |
| 30 | | Outcomes After Stereotactic Body Radiotherapy or Radiofrequency Ablation for Hepatocellular Carcinoma | FENG, M | J. Clin. Oncol. | 2016 | 253 | 45.313 |  |
| 31 | | Systemic review of the patterns of failure following stereotactic body radiation therapy in early-stage non-small-cell lung cancer: Clinical implications | CHI, A | Radiother. Oncol. | 2010 | 250 | 21.429 |  |
| 32 | | Outcomes of Stereotactic Ablative Radiotherapy in Patients With Potentially Operable Stage I Non-Small Cell Lung Cancer | LAGERWAARD, FJ | Int. J. Radiat. Oncol. Biol. Phys. | 2012 | 248 | 26.571 |  |
| 33 | | Phase 2 Multi-institutional Trial Evaluating Gemcitabine and Stereotactic Body Radiotherapy for Patients With Locally Advanced Unresectable Pancreatic Adenocarcinoma | HERMAN, JM | Cancer | 2015 | 238 | 37.091 |  |
| 34 | | Effect of Pembrolizumab After Stereotactic Body Radiotherapy vs Pembrolizumab Alone on Tumor Response in Patients With Advanced Non-Small Cell Lung Cancer: Results of the PEMBRO-RT Phase 2 Randomized Clinical Trial | THEELEN, WSME | JAMA Oncol. | 2019 | 228 | 114 |  |
| 35 | | Safety and Clinical Activity of Pembrolizumab and Multisite Stereotactic Body Radiotherapy in Patients With Advanced Solid Tumors | CHMURA, SJ | J. Clin. Oncol. | 2018 | 228 | 70.154 |  |
| 36 | | STEREOTACTIC BODY RADIOTHERAPY FOR LOCALIZED PROSTATE CANCER: INTERIM RESULTS OF A PROSPECTIVE PHASE II CLINICAL TRIAL | KING, CR | Int. J. Radiat. Oncol. Biol. Phys. | 2009 | 227 | 17.921 |  |
| 37 | | Promising clinical outcome of stereotactic body radiation therapy for patients with inoperable Stage I/II non-small-cell lung cancer | SALAMA, JK | Int. J. Radiat. Oncol. Biol. Phys. | 2006 | 224 | 14.933 |  |
| 38 | | Stereotactic body radiotherapy for multisite extracranial oligometastases Final report of a dose escalation trial in patients with 1 to 5 sites of metastatic disease | XIA, TY | Cancer | 2012 | 223 | 23.069 |  |
| 39 | | Prospective Trial of Stereotactic Body Radiation Therapy for Both Operable and Inoperable T1N0M0 Non-Small Cell Lung Cancer: Japan Clinical Oncology Group Study JCOG0403 | NAGATA, Y | Int. J. Radiat. Oncol. Biol. Phys. | 2015 | 221 | 38.435 |  |
| 40 | | Stereotactic body radiation therapy: a novel treatment modality | LO, SS | Nat. Rev. Clin. Oncol. | 2010 | 215 | 18.429 |  |
| 41 | | Gemcitabine chemotherapy and single-fraction stereotactic body radiotherapy for locally advanced pancreatic cancer | KOONG, AC | Int. J. Radiat. Oncol. Biol. Phys. | 2008 | 215 | 16.753 |  |
| 42 | | Stereotactic Body Radiation Therapy for Locally Advanced and Borderline Resectable Pancreatic Cancer Is Effective and Well Tolerated | SHRIDHAR, R | Int. J. Radiat. Oncol. Biol. Phys. | 2013 | 213 | 26.082 |  |
| 43 | | Clinical outcomes of 3D conformal hypofractionated single high-dose radiotherapy for one or two lung tumors using a stereotactic body frame | NAGATA, Y | Int. J. Radiat. Oncol. Biol. Phys. | 2002 | 213 | 10.923 |  |
| 44 | | Phase 1 Study of Stereotactic Body Radiotherapy and Interleukin-2: Tumor and Immunological Responses | CURTI, BD | Sci. Transl. Med. | 2012 | 211 | 22.811 |  |
| 45 | | Stereotactic body radiation therapy for early-stage non-small cell lung cancer: Executive Summary of an ASTRO Evidence-Based Guideline | VIDETIC, GMM | Pract. Radiat. Oncol. | 2017 | 206 | 51.5 |  |
| 46 | | Stereotactic body radiation therapy for early stage non-small cell lung cancer: Results of a prospective trial | RICARDI, U | Lung Cancer | 2010 | 203 | 17.781 |  |
| 47 | | Phase I feasibility trial of stereotactic body radiation therapy for primary hepatocellular carcinoma | CARDENES, HR | Clin. Transl. Oncol. | 2010 | 203 | 17.652 |  |
| 48 | | DOSE-ESCALATION STUDY OF SINGLE-FRACTION STEREOTACTIC BODY RADIOTHERAPY FOR LIVER MALIGNANCIES | GOODMAN, KA | Int. J. Radiat. Oncol. Biol. Phys. | 2010 | 202 | 18.504 |  |
| 49 | | Stage I-II non-small-cell lung cancer treated using either stereotactic ablative radiotherapy (SABR) or lobectomy by video-assisted thoracoscopic surgery (VATS): outcomes of a propensity score-matched analysis | VERSTEGEN, NE | Ann. Oncol. | 2013 | 194 | 23.515 |  |
| 50 | | Stereotactic body radiation therapy for inoperable hepatocellular carcinoma as a local salvage treatment after incomplete transarterial chemoembolization | KIM, MS | Cancer | 2012 | 190 | 21.509 |  |
| 51 | | STEREOTACTIC BODY RADIATION THERAPY IN CENTRALLY AND SUPERIORLY LOCATED STAGE I OR ISOLATED RECURRENT NON-SMALL-CELL LUNG CANCER | CHANG, JY | Int. J. Radiat. Oncol. Biol. Phys. | 2008 | 190 | 14.805 |  |
| 52 | | FRAMELESS STEREOTACTIC BODY RADIOTHERAPY FOR LUNG CANCER USING FOUR-DIMENSIONAL CONE BEAM CT GUIDANCE | SONKE, JJ | Int. J. Radiat. Oncol. Biol. Phys. | 2009 | 187 | 15.265 |  |
| 53 | | A prospective pilot study of curative-intent stereotactic body radiation therapy in patients with 5 or fewer oligometastatic lesions | WULF, J | Cancer | 2008 | 186 | 13.693 |  |
| 54 | | Stereotactic radiotherapy of extracranial targets: CT-simulation and accuracy of treatment in the stereotactic body frame | MILANO, MT | Radiother. Oncol. | 2000 | 186 | 8.928 |  |
| 55 | | Phase II Trial of Stereotactic Body Radiation Therapy Combined With Erlotinib for Patients With Limited but Progressive Metastatic Non-Small-Cell Lung Cancer | TIMMERMAN, R | J. Clin. Oncol. | 2014 | 184 | 27.259 |  |
| 56 | | Vertebral Compression Fracture After Spine Stereotactic Body Radiotherapy: A Multi-Institutional Analysis With a Focus on Radiation Dose and the Spinal Instability Neoplastic Score | SAHGAL, A | J. Clin. Oncol. | 2013 | 184 | 23 |  |
| 57 | | Stereotactic body radiation therapy for management of spinal metastases in patients without spinal cord compression: a phase 1-2 trial | WANG, XS | Lancet Oncol. | 2012 | 184 | 19.54 |  |
| 58 | | Stereotactic Body Radiotherapy for Colorectal Liver Metastases A Pooled Analysis | CHANG, DT | Cancer | 2011 | 180 | 18 |  |
| 59 | | Outcomes of Observation vs Stereotactic Ablative Radiation for Oligometastatic Prostate Cancer The ORIOLE Phase 2 Randomized Clinical Trial | TRAN, PT | JAMA Oncol. | 2020 | 178 | 133.5 |  |
| 60 | | A Survey of Stereotactic Body Radiotherapy Use in the United States | LAWSON, JD | Cancer | 2011 | 178 | 17.95 |  |
| 61 | | Progression-free Survival Following Stereotactic Body Radiotherapy for Oligometastatic Prostate Cancer Treatment-naive Recurrence: A Multi-institutional Analysis | OST, P | Eur. Urol. | 2016 | 177 | 31.235 |  |
| 62 | | Outcomes of stereotactic ablative radiotherapy for central lung tumours: A systematic review | SENTHI, S | Radiother. Oncol. | 2013 | 177 | 20.824 |  |
| 63 | | Stereotactic body radiation therapy versus surgical resection for stage I non-small cell lung cancer | CRABTREE, TD | J. Thorac. Cardiovasc. Surg. | 2010 | 176 | 15.88 |  |
| 64 | | Factors important for efficacy of stereotactic body radiotherapy of medically inoperable stage I lung cancer. A retrospective analysis of patients treated in the Nordic countries | LEWENSOHN, R | Acta Oncol. | 2006 | 175 | 11.667 |  |
| 65 | | Hypofractionated stereotactic body radiation therapy (SBRT) for limited hepatic metastases | KATZ, AW | Int. J. Radiat. Oncol. Biol. Phys. | 2007 | 174 | 12 |  |
| 66 | | Stereotactic body radiotherapy for low-risk prostate cancer: five-year outcomes | KING, CHRISTOPHER R | Radiat. Oncol. | 2011 | 172 | 16.125 |  |
| 67 | | Long-term effect of stereotactic body radiation therapy for primary hepatocellular carcinoma ineligible for local ablation therapy or surgical resection. Stereotactic radiotherapy for liver cancer | BAE, SH | BMC Cancer | 2010 | 172 | 15.636 |  |
| 68 | | Outcomes of Stereotactic Ablative Radiotherapy for Centrally Located Early-Stage Lung Cancer | HAASBEEK, CJA | J. Thorac. Oncol. | 2011 | 171 | 17.538 |  |
| 69 | | Repeated stereotactic body radiotherapy for oligometastatic prostate cancer recurrence | OST, P | Radiat. Oncol. | 2014 | 168 | 23.172 |  |
| 70 | | CHEST WALL VOLUME RECEIVING > 30 GY PREDICTS RISK OF SEVERE PAIN AND/OR RIB FRACTURE AFTER LUNG STEREOTACTIC BODY RADIOTHERAPY | LARNER, JM | Int. J. Radiat. Oncol. Biol. Phys. | 2010 | 168 | 14.609 |  |
| 71 | | A Randomized Phase 2 Study Comparing 2 Stereotactic Body Radiation Therapy Schedules for Medically Inoperable Patients With Stage I Peripheral Non-Small Cell Lung Cancer: NRG Oncology RTOG 0915 (NCCTG N0927) | VIDETIC, GMM | Int. J. Radiat. Oncol. Biol. Phys. | 2015 | 167 | 28.629 |  |
| 72 | | Ipilimumab with Stereotactic Ablative Radiation Therapy: Phase I Results and Immunologic Correlates from Peripheral T Cells | TANG, C; WELSH, JW | Clin. Cancer Res. | 2017 | 165 | 36.667 |  |
| 73 | | Immunotherapy and stereotactic ablative radiotherapy (ISABR): a curative approach? | CHANG, JY | Nat. Rev. Clin. Oncol. | 2016 | 165 | 32.459 |  |
| 74 | | Phase I Dose-Escalation Study of Stereotactic Body Radiation Therapy for Low- and Intermediate-Risk Prostate Cancer | TIMMERMAN, R | J. Clin. Oncol. | 2011 | 165 | 15.968 |  |
| 75 | | Stereotactic body radiation therapy (SBRT) for lung metastases | OKUNIEFF, P | Acta Oncol. | 2006 | 163 | 10.867 |  |
| 76 | | Stereotactic body radiation therapy - A comprehensive review | CHANG, BK | Am. J. Clin. Oncol.-Cancer Clin. Trials | 2007 | 162 | 11.782 |  |
| 77 | | Stereotactic Body Radiation Therapy (SBRT) for clinically localized prostate cancer: the Georgetown University experience | COLLINS, SP | Radiat. Oncol. | 2013 | 158 | 18.588 |  |
| 78 | | SPACE - A randomized study of SBRT vs conventional fractionated radiotherapy in medically inoperable stage I NSCLC | NYMAN, J | Radiother. Oncol. | 2016 | 157 | 31.932 |  |
| 79 | | Lobectomy, Sublobar Resection, and Stereotactic Ablative Radiotherapy for Early-Stage Non-Small Cell Lung Cancers in the Elderly | SMITH, BD | JAMA Surg. | 2014 | 155 | 22.963 |  |
| 80 | | American Society for Therapeutic Radiology and Oncology and American College of Radiology Practice Guideline for the Performance of Stereotactic Body Radiation Therapy | PALMA, DA | Int. J. Radiat. Oncol. Biol. Phys. | 2004 | 154 | 9.1485 |  |
| 81 | | Radiographic changes after lung stereotactic ablative radiotherapy (SABR) - Can we distinguish recurrence from fibrosis? A systematic review of the literature | BALL, D | Radiother. Oncol. | 2012 | 152 | 16 |  |
| 82 | | Stereotactic body radiotherapy for localized prostate cancer: disease control and quality of life at 6 years | KATZ, AJ | Radiat. Oncol. | 2013 | 151 | 18.12 |  |
| 83 | | STEREOTACTIC BODY RADIOTHERAPY AND GEMCITABINE FOR LOCALLY ADVANCED PANCREATIC CANCER | MAHADEVAN, A | Int. J. Radiat. Oncol. Biol. Phys. | 2010 | 151 | 13.938 |  |
| 84 | | STEREOTACTIC BODY RADIOTHERAPY IS EFFECTIVE SALVAGE THERAPY FOR PATIENTS WITH PRIOR RADIATION OF SPINAL METASTASES | SAHGAL, A | Int. J. Radiat. Oncol. Biol. Phys. | 2009 | 151 | 12.411 |  |
| 85 | | Stereotactic body radiotherapy for oligometastatic lung tumors | NAGATA, Y | Int. J. Radiat. Oncol. Biol. Phys. | 2008 | 151 | 11.69 |  |
| 86 | | Stereotactic ablative radiotherapy versus standard radiotherapy in stage 1 non-small-cell lung cancer (TROG 09.02 CHISEL): a phase 3, open-label, randomised controlled trial | KONG, FMS | Lancet Oncol. | 2019 | 150 | 62.069 |  |
| 87 | | Survival Outcome After Stereotactic Body Radiation Therapy and Surgery for Stage I Non-Small Cell Lung Cancer: A Meta-Analysis | CHANG, JY | Int. J. Radiat. Oncol. Biol. Phys. | 2014 | 149 | 21.805 |  |
| 88 | | Stereotactic Ablative Radiation Therapy for Centrally Located Early Stage or Isolated Parenchymal Recurrences of Non-Small Cell Lung Cancer: How to Fly in a No Fly Zone | FAKIRIS, AJ | Int. J. Radiat. Oncol. Biol. Phys. | 2014 | 149 | 20.09 |  |
| 89 | | SINGLE-FRACTION STEREOTACTIC BODY RADIATION THERAPY AND SEQUENTIAL GEMCITABINE FOR THE TREATMENT OF LOCALLY ADVANCED PANCREATIC CANCER | KOONG, AC | Int. J. Radiat. Oncol. Biol. Phys. | 2011 | 147 | 14.7 |  |
| 90 | | DOSE-RESPONSE RELATIONSHIP FOR IMAGE-GUIDED STEREOTACTIC BODY RADIOTHERAPY OF PULMONARY TUMORS: RELEVANCE OF 4D DOSE CALCULATION | GUCKENBERGER, M | Int. J. Radiat. Oncol. Biol. Phys. | 2009 | 147 | 11.919 |  |
| 91 | | Stereotactic body radiotherapy for small hepatocellular carcinoma: A retrospective outcome analysis in 185 patients | KUNIEDA, E | Acta Oncol. | 2014 | 146 | 19.467 |  |
| 92 | | A DOSE-VOLUME ANALYSIS OF RADIATION PNEUMONITIS IN NON-SMALL CELL LUNG CANCER PATIENTS TREATED WITH STEREOTACTIC BODY RADIATION THERAPY | SAHGAL, A | Int. J. Radiat. Oncol. Biol. Phys. | 2012 | 146 | 15.103 |  |
| 93 | | SPINAL CORD TOLERANCE FOR STEREOTACTIC BODY RADIOTHERAPY | KOTO, M | Int. J. Radiat. Oncol. Biol. Phys. | 2010 | 145 | 12.889 |  |
| 94 | | A phase II study on stereotactic body radiotherapy for stage I non-small cell lung cancer | BERKOVIC, P | Radiother. Oncol. | 2007 | 145 | 10.545 |  |
| 95 | | Salvage Stereotactic Body Radiotherapy for Patients With Limited Prostate Cancer Metastases: Deferring Androgen Deprivation Therapy | SAHGAL, A | Clin. Genitourin. Cancer | 2013 | 144 | 16.941 |  |
| 96 | | Vertebral Compression Fracture (VCF) After Spine Stereotactic Body Radiation Therapy (SBRT): Analysis of Predictive Factors | MCCAMMON, R | Int. J. Radiat. Oncol. Biol. Phys. | 2012 | 144 | 16.302 |  |
| 97 | | OBSERVATION OF A DOSE-CONTROL RELATIONSHIP FOR LUNG AND LIVER TUMORS AFTER STEREOTACTIC BODY RADIATION THERAPY | SAHGAL, A | Int. J. Radiat. Oncol. Biol. Phys. | 2009 | 142 | 11.211 |  |
| 98 | | REIRRADIATION HUMAN SPINAL CORD TOLERANCE FOR STEREOTACTIC BODY RADIOTHERAPY | SAHGAL, A | Int. J. Radiat. Oncol. Biol. Phys. | 2012 | 141 | 14.586 |  |
| 99 | | Probabilities of Radiation Myelopathy Specific to Stereotactic Body Radiation Therapy to Guide Safe Practice | KAVANAGH, BD | Int. J. Radiat. Oncol. Biol. Phys. | 2013 | 139 | 16.194 |  |
| 100 | | Interim analysis of a prospective phase I/II trial of SBRT for liver metastases | POTTERS, L | Acta Oncol. | 2006 | 138 | 9.2 |  |
